# The Milan System SUMP Category: 5‐Year Diagnostic Performance

**DOI:** 10.1002/dc.25482

**Published:** 2025-05-06

**Authors:** Henri Lagerstam, Erkka Tommola, David Kalfert, Saara Kares, Heini Huhtala, Ivana Kholová

**Affiliations:** ^1^ Faculty of Medicine and Health Technology, Tampere University Tampere Finland; ^2^ Fimlab Laboratories Pathology Tampere Finland; ^3^ Department of Otorhinolaryngology and Head and Neck Surgery, First Faculty of Medicine University Hospital Motol, Charles University Prague Czech Republic; ^4^ Faculty of Social Sciences, Tampere University Tampere Finland; ^5^ Institute of Clinical Medicine, Pathology and Forensic Medicine University of Eastern Finland Kuopio Finland; ^6^ Department of Clinical Pathology Diagnostic Imaging Center, Kuopio University Hospital Kuopio Finland

**Keywords:** fine‐needle aspiration, Milan system for reporting salivary cytopathology, salivary gland, salivary gland neoplasm of uncertain malignant potential, SUMP

## Abstract

**Background:**

Salivary gland neoplasms are heterogeneous, with wide cytomorphological overlap. Neoplasms categorized in the Milan System for Reporting Salivary Gland Cytopathology (MSRSGC) as salivary gland neoplasms of uncertain malignant potential (SUMP) reflect this diagnostic challenge.

**Methods:**

All salivary gland fine‐needle aspirations (SG‐FNA) diagnosed at Fimlab Laboratories over a 5‐year period (January 1, 2018–December 31, 2022) that were classified as SUMP were included. Follow‐ups were reviewed until May 31, 2024. The SUMP cases were classified into cytomorphological subtypes. The risk of malignancy (ROM), risk of neoplasm, and median timelines of the pathology laboratory workflow and patient management were calculated. In addition, separate analyses of the impact of age and sex were performed.

**Results:**

A total of 1157 SG‐FNAs were diagnosed over a 5‐year period. Of these, 100 SG‐FNAs from 52 females and 33 males were classified as SUMP. A total of 69 (69.0%) SUMP cases underwent histological verification. The ROM was 23.2% for all surgical follow‐ups, 35.0% for the oncocytic/oncocytoid subtype, and 18.4% for the basaloid subtype. For the male and female groups, the ROMs were 26.9% and 16.2% for all surgical follow‐ups, 40.0% and 14.3% for the oncocytic/oncocytoid subtype, and 18.8% and 16.7% for the basaloid subtype, respectively.

**Conclusions:**

The 5‐year analysis of SUMP category performance showed a lower ROM in our practice than the MSRSGC reference value. The oncocytic/oncocytoid subtype presented a higher ROM than the basaloid subtype. The ROMs were highest in the male population and in the age group of 30–69 years.

## 1 Introduction

Salivary gland tumors represent only 3%–6% of all head and neck tumors. In addition to the low incidence of salivary gland neoplasms, the cytomorphology of salivary gland tumors is challenging. This makes salivary gland cytopathology one of the most difficult cytopathology diagnostic areas [1, 2]. Salivary gland tumors are more common in the older population. Most salivary gland tumors occur in people over 40 years of age, and the risk of malignancy also increases with age. The risk of both benign and malignant salivary gland tumors is highest in the sixth and seventh decades of life. Salivary gland tumors are slightly more common in the female population [3, 4].

The second edition of the Milan System for Reporting Salivary Gland Cytopathology (MSRSGC) [5] was published in 2023, 5 years after the first edition [6]. The MSRSGC was issued to address the absence of a coherent system for categorizing salivary gland fine‐needle aspiration (SG‐FNA) specimens. The MSRSGC consists of six diagnostic categories: (1) non‐diagnostic (ND), (2) non‐neoplastic (NN), (3) atypia of undetermined significance (AUS), (4a) benign neoplasm (BN), (4b) salivary gland neoplasm of uncertain malignant potential (SUMP), (5) suspicious for malignancy (SM), and (6) malignant neoplasm (MN).

SUMP is one of the most challenging categories of the MSRSGC because of its heterogeneity. Specimens that are neoplastic but have both benign and malignant neoplastic features or specimens that meet the characteristics of benign tumors but have abnormal cell components or clinical findings suggesting malignancy should be categorized as SUMP. The MSRSGC reference risk of malignancy (ROM) value for the SUMP category is 35%. The risk of neoplasm (RON) should be close to 100% due to the neoplastic characteristics of the SUMP category; nevertheless, it is not officially estimated in the MSRSGC [5].

Delays in the diagnostic process and patient management can reduce life expectancy. The timelines from SG‐FNA to surgical resection depend on the MSRSGC classification. Average timelines are the shortest in the categories with the highest risk of malignancy, that is, SM and MN, approximately 30 days [7, 8]. The SUMP category also leads to rapid surgical resection (approximately 50–60 days), as the MSRSGC also recommends surgery in the SUMP category [5].

During the Covid‐19 pandemic, diagnostic procedures and patient management were delayed. Elective procedures such as SG‐FNAs were delayed. Due to prioritization, the number of malignant cases increased during the Covid‐19 pandemic [9, 10]. The present study evaluates a 5‐year (2018–2022) analysis of SUMP category diagnostic outcomes in a tertiary care center, including an analysis of the prevalence of sex and age and cytomorphological subtyping.

## 2 Materials and Methods

A search was carried out on the laboratory information system database to find all SG‐FNAs diagnosed at the Department of Pathology, Fimlab Laboratories, Tampere, Finland. Samples were taken at Tampere University Hospital, regional hospitals, and community health care centers. The MSRSGC was used to categorize all SG‐FNAs over a 5‐year period (January 1, 2018–December 31, 2022). All SG‐FNAs classified as SUMP were included in the study cohort. Histological follow‐ups of SUMP cases were checked until May 31, 2024.

Radiologists used ultrasound‐guided FNAs with a 22G needle in all cases. No rapid‐on‐site evaluation (ROSE) was performed. Preparations were alcohol‐fixed, cytospun, and stained with Papanicolaou stain. Four experienced pathologists with special interest in cytopathology and head and neck surgical pathology signed out cases. Cell block (CB) was prepared in all cases after material was triaged and the following CB methods were used: in‐house method, plasma‐thrombin, collection of visible fragments, and Shandon cytoblock kit (Thermo Fisher Scientific, Waltham, MA, USA) [11, 12, 13]. Immunohistochemistry was applied to CB at the discretion of the signing‐out pathologist.

The patients' age, sex, and lesion topography and size were recorded in Microsoft Excel for Microsoft 365 MSO (Version 2111 Build 16.0.14701.20254) 64‐bit. Histological diagnoses were also tabulated, if available. Separate sample‐ and patient‐based analyses were performed. The SUMP cases were subclassified into oncocytic/oncocytoid and basaloid subtypes, and the ROM and RON were calculated. For both ROM and RON, we calculated the upper‐ and lower‐bound risks. Lower‐bound ROM was calculated by dividing malignant cases by all cases, and upper‐bound ROM was calculated by dividing malignant cases by histologically confirmed cases. The lower‐bound RON was calculated by dividing all cases of neoplasms by all cases, and the upper‐bound RON was calculated using histologically verified cases as the denominator.

The dates of FNA and surgical procedures were recorded, as were the dates of pathology laboratory workflows, and the timelines were calculated. Six different median timelines were calculated in days and quartiles (min/max): (1) the timeline from the FNA procedure to the pathology laboratory SUMP diagnosis, (2) the timeline from the surgical procedure to the pathology laboratory final histological diagnosis, (3) the timeline from the first FNA with the SUMP diagnosis to the first repeated FNA, (4) the timeline from the first FNA with the SUMP diagnosis to surgical management, (5) the timeline from the last FNA with the SUMP diagnosis to surgical management, and (6) the timeline from the last FNA to surgical management. In addition, we conducted sex and age analyses: (1) female versus male cohort and (2) study cohort divided into three age categories: 20–29 years old, 30–69 years old, and 70+ years old.

This study was conducted in accordance with the Helsinki Declaration, and the Pirkanmaa Hospital District Ethical Committee approved the use of the material (R17174). Individual consent was not required for the study.

Statistical analysis was performed using IBM SPSS Statistics (version 22.0; SPSS, IBM, Armonk, NY, USA). Data were analyzed using descriptive statistics, the Mann–Whitney *U* test, and the Fisher exact test; *p* values equal to or less than 0.05 were considered significant.

## 3 Results

### 3.1 Study Cohort

In a 5‐year period, a total of 1157 SG‐FNAs were diagnosed at our institution. SUMP was the cytological diagnosis in 100 (8.6%) samples from 85 patients. Of these patients, 52 (61.2%) were female and 33 (38.8%) were male. Of the SUMP cases, 67 were subcategorized as basaloid and 33 as oncocytic/oncocytoid. Topographically, 89 cases were taken from the parotid gland and 11 from the submandibular gland. The characteristics of the study cohort are shown in Table 1.

**TABLE 1:** Summary of characteristics of the study cohort in sample‐based and patient‐based analyses.

Analysis type	Grouping	Total	Sex		Median age in years (range)	Lesion location		Average lesion size ± SD (cm)
			Female, *n* (%)	Male, *n* (%)		Parotid gland (%)	Submandibular gland (%)	
Sample‐based	All samples	100	64 (64.0)	36 (36.0)	72.5 (20–96)	89 (89.0)	11 (11.0)	2.0 ± 1.1
	Samples with surgical follow‐up	69	41 (59.4)	28 (40.6)	67 (20–91)	59 (85.5)	10 (14.5)	1.9 ± 1.1
Patient‐based	All patients	85	52 (61.2)	33 (38.8)	71 (20–96)	75 (88.2)	10 (11.8)	2.0 ± 1.1
	Patients with surgical follow‐up	63	37 (58.7)	26 (41.3)	67 (20–91)	54 (85.7)	9 (14.3)	2.0 ± 1.1

### 3.2 Histological Follow‐Ups

**FIGURE 1:**
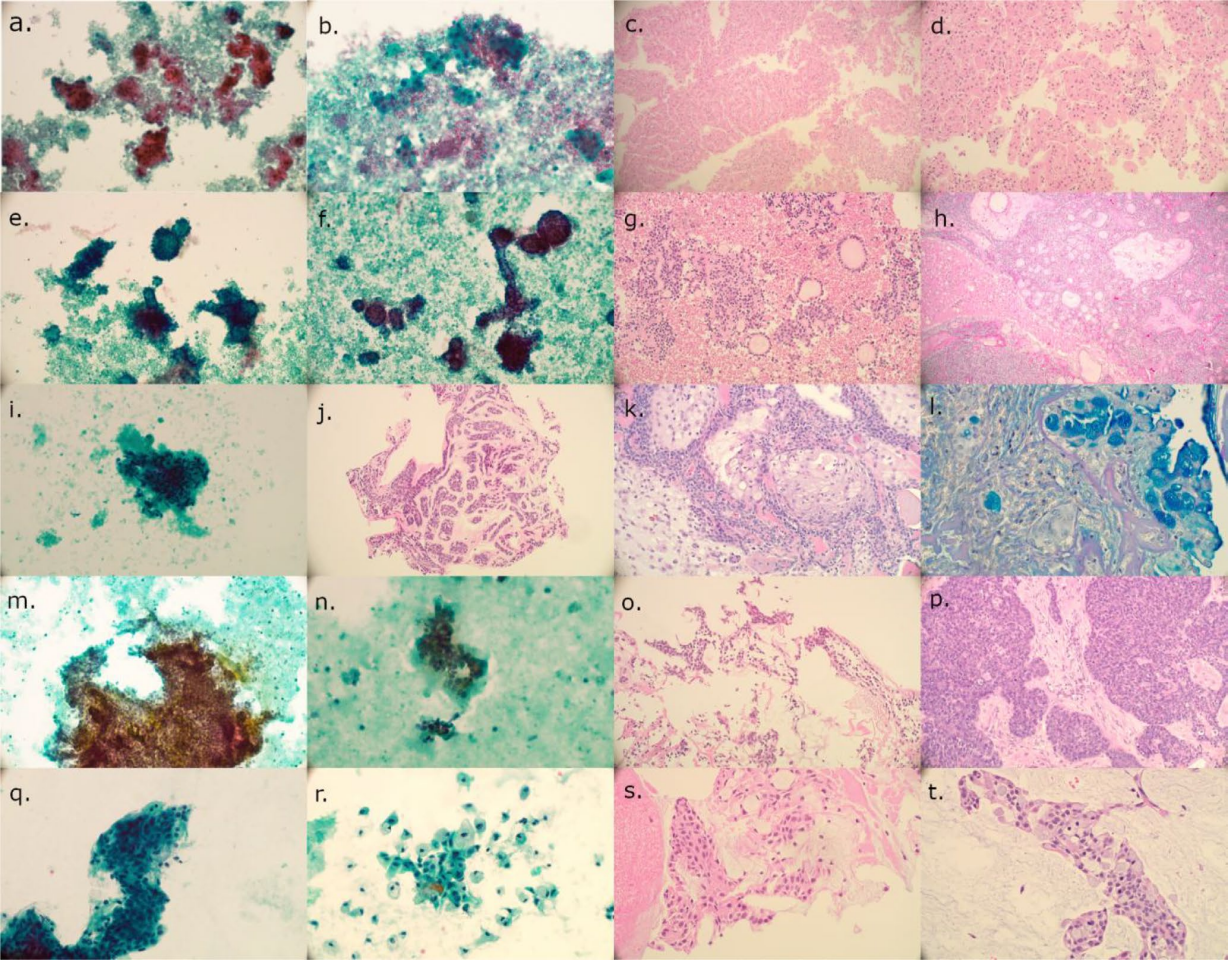
Histologically verified SUMP cases from our cohort. (a) Oncocytic/oncocytoid subtype of SUMP with oncocytic cells in small group. Note bloody background. Papanicolaou stain, original magnification ×200. (b) Oncocytic/oncocytoid subtype of SUMP with oncocytic cells in small group and bloody background. Same case (a). Papanicolaou stain, original magnification ×400. (c) Cell block made from fine‐needle aspirate presented in (a) and (b). H&E stain, original magnification ×100. (d) Cell block made from fine‐needle aspirate presented in (a) and (b). Nodular oncocytic hyperplasia was the histological diagnosis. H&E stain, original magnification ×200. (e) Basaloid subtype of SUMP with basaloid cells in 3D balls and trabeculae. Papanicolaou stain, original magnification ×200. (f) Basaloid tumor as shown in (e). Papanicolaou stain, original magnification ×200. (g) Cell block revealed two populations of basaloid cells. Same case as (e) and (f). H&E stain, original magnification ×200. (h) Histological specimen revealed basal cell adenoma. Same case as (e–g). PAS, original magnification ×200. (i) Sparse cytospin sample with some epithelioid mildly atypical cells. No stroma was present. Papanicolaou stain, original magnification ×400. (j) Cellular rich cell block showed some trabeculae and sparse ducts revealing biphasic histology. Same case as (i). H&E stain, original magnification ×200. (k) Histological specimen of (i, j). Case showed pleomorphic adenoma with large squamous cell metaplasia. H&E stain, original magnification ×200. (l) Histological specimen showed pleomorphic adenoma with mucinous metaplasia that caused SUMP upgrade in addition to mild atypia. Alcian Blue‐PAS, original magnification ×400. (m) Epithelial fragment with nuclear size and shape variability. Sparse stroma is present. Papanicolaou stain, original magnification ×200. (n) Details of moderately atypical cells from same case as (m). Papanicolaou stain, original magnification ×400. (o) Mildly cellular cell block from (m, n) case. H&E stain, original magnification ×200. (p) Histological verification of (m–o). Case showed carcinoma ex pleomorphic adenoma. H&E stain, original magnification ×200. (q) Papillary fragment formed by intermediate and mucinous cells. Papanicolaou stain, original magnification ×400. (r) Mixture of intermediate and mucinous cells in cytospin sample. Same case as in (q). Papanicolaou stain, original magnification ×400. (s) Cell block showed intermixed mucinous cells and intermediate cells. H&E stain, original magnification ×400. (t) Histological diagnosis was mucoepidermoid carcinoma in (q–s) case. H&E stain, original magnification ×200. [Color figure can be viewed at wileyonlinelibrary.com]

Of all samples diagnosed as SUMP, 21 (21.0%) were diagnosed in 2018, 16 (16.0%) in 2019, 13 (13.0%) in 2020, 23 (23.0%) in 2021, and 27 (27.0%) in 2022. A total of 69 (69.0%) SUMP cases underwent histological follow‐up. Histopathological diagnoses were non‐neoplastic in 5 (7.2%) cases, 48 (69.6%) cases were diagnosed as benign neoplasms, and only 16 (23.2%) were malignant neoplasms. Non‐neoplastic cases were nodular oncocytic hyperplasia in three cases and chronic sialadenitis in two cases. Pleomorphic adenoma was the most common benign neoplasm (*n* = 24), and carcinoma ex pleomorphic adenoma (*n* = 5) was the most common malignant neoplasm. All histopathological diagnoses are listed in Table 2 and a microphotograph panel of neoplasms is shown in Figure 1.

**TABLE 2:** Sample‐based histopathological diagnoses in salivary gland neoplasm of uncertain malignant potential category cases with surgical follow‐up and patient‐based according to the age and sex.

	Non‐neoplastic, *n* = 5 (7.2%)	Benign neoplasm, *n* = 48 (69.6%)	Malignant neoplasm, *n* = 16 (23.2%)
All cases (sample‐based)	Nodular oncocytic hyperplasia (*n* = 3) Chronic sialadenitis (*n* = 2)	Pleomorphic adenoma (*n* = 24) Basal cell adenoma (*n* = 6) Warthin's tumor (*n* = 6) Myoepithelioma (*n* = 5) Cystadenoma (*n* = 3) Oncocytoma (*n* = 3) Eccrine spiradenoma (*n* = 1)	Carcinoma ex pleomorphic adenoma (*n* = 5) Mucoepidermoid carcinoma (*n* = 4) Adenoid cystic carcinoma (*n* = 3) Acinic cell carcinoma (*n* = 2) Secretory carcinoma (*n* = 1) Epithelial‐myoepithelial carcinoma (*n* = 1)
Oncytic/oncocytoid (smaple‐based)	Nodular oncocytic hyperplasia (*n* = 2)	Warthin's tumor (*n* = 6) Oncocytoma (*n* = 3) Cystadenoma (*n* = 2)	Mucoepidermoid carcinoma (*n* = 4) Acinic cell carcinoma (*n* = 2) Secretory carcinoma (*n* = 1)
Basaloid (sample‐based)	Chronic sialadenitis (*n* = 2) Nodular oncocytic hyperplasia (*n* = 1)	Pleomorphic adenoma (*n* = 24) Basal cell adenoma (*n* = 6) Myoepithelioma (*n* = 5) Cystadenoma (*n* = 1) Eccrine spiradenoma (*n* = 1)	Carcinoma ex pleomorphic adenoma (*n* = 5) Adenoid cystic carcinoma (*n* = 3) Epithelial‐myoepithelial carcinoma (*n* = 1)
20–29 years old (female/male) (patient‐based)		Pleomorphic adenoma (2F/2M) Myoepithelioma (1F)	
30–69 years old (female/male) (patient‐based)	Nodular oncocytic hyperplasia (1F) Chronic sialadenitis (1F)	Pleomorphic adenoma (8F/4M) Warthin's tumor (1F/3M) Myoepithelioma (2F/1M) Basal cell adenoma (1F/1M) Oncocytoma (1F)	Carcinoma ex pleomorphic adenoma (1F/3M) Acinic cell carcinoma (1M) Adenoid cystic carcinoma (1F) Epithelial‐myoepithelial carcinoma (1F) Secretory carcinoma (1M)
70+ years old (female/male) (patient based)	Nodular oncocytic hyperplasia (1F) Chronic sialadenitis (1F)	Pleomorphic adenoma (4F/4M) Basal cell adenoma (3F) Cystadenoma (3M) Warthin's tumor (1F/1M) Eccrine spiradenoma (1F) Myoepithelioma (1F) Oncocytoma (1F)	Mucoepidermoid carcinoma (1F/2M) Adenoid cystic carcinoma (1F) Carcinoma ex plemorphic adenoma (1F)

### 3.3 ROM, RON, and Subtypes

Of the histologically verified cases, 16 were malignant. Upper‐bound ROM was 23.2%, and lower‐bound ROM was 16.0%. The basaloid subtype showed 18.4% upper‐bound ROM, and the oncocytic/oncocytoid subtype showed 35.0% ROM (*p* = 0.207). Five‐year patient‐ and sample‐based and subcategory analyses are shown in Table 3.

**TABLE 3:** Sample‐ and patient‐based, and subcategory analyses of salivary gland neoplasm of uncertain malignant potential category in a 5‐year period (2018–2022).

Analysis	No. of cases	Cases with surgical follow‐up, *n* (%)	Histological diagnoses			Upper bound ROM (%)	Lower bound ROM (%)	Upper bound RON (%)	Lower bound RON (%)
			NN	BN	MN				
Sample‐based	100	69 (69.0)	5	48	16	23.2	16.0	92.8	64.0
Basaloid sample	67	49 (73.1)	3	37	9	18.4	13.4	93.9	68.7
Oncocytic/oncocytoid sample	33	20 (60.6)	2	11	7	35.0	21.2	90.0	54.5
Patient‐based	85	63 (74.1)	4	46	13	20.6	15.3	93.7	69.4
20–29 years old	5	5 (100.0)	0	5	0	0.0	0.0	100.0	100.0
30–69 years old	36	32 (88.9)	2	22	8	25.0	22.2	93.8	83.3
70+ years old	44	26 (59.1)	2	19	5	19.2	11.4	92.3	54.5
Female	52	37 (71.2)	4	27	6	16.2	11.5	89.2	63.5
Male	33	26 (78.8)	0	19	7	26.9	21.2	100.0	78.8
Basaloid 20–29 years old	5	5 (100.0)	0	5	0	0.0	0.0	100.0	100.0
Basaloid 30–69 years old	28	25 (89.3)	2	17	6	24.0	21.4	92.0	82.1
Basaloid 70+ years old	25	17 (68.0)	1	14	2	11.8	8.0	94.1	64.0
Basaloid female	40	30 (75.0)	2	23	5	16.7	12.5	93.3	70.0
Basaloid male	17	16 (94.1)	0	13	3	18.8	17.6	100.0	94.1
Oncocytic/oncocytoid 20–29 years old	0	0	0	0	0	N.D.	N.D.	N.D.	N.D.
Oncocytic/oncocytoid 30–69 years old	8	7 (87.5)	0	5	2	28.6	25.0	100.0	87.5
Oncocytic/oncocytoid 70+ years old	19	9 (47.4)	1	5	3	33.3	15.8	88.9	42.1
Oncocytic/oncocytoid female	12	7 (58.3)	0	6	1	14.3	8.3	100.0	58.3
Oncocytic/oncocytoid male	16	10 (62.5)	0	6	4	40.0	25.0	100.0	62.5

Abbreviations: BN, benign neoplasm; MN, malignant neoplasm; N.D., not determined; NN, non‐neoplastic; ROM, risk of malignancy; RON, risk of neoplasm.

**FIGURE 2:**
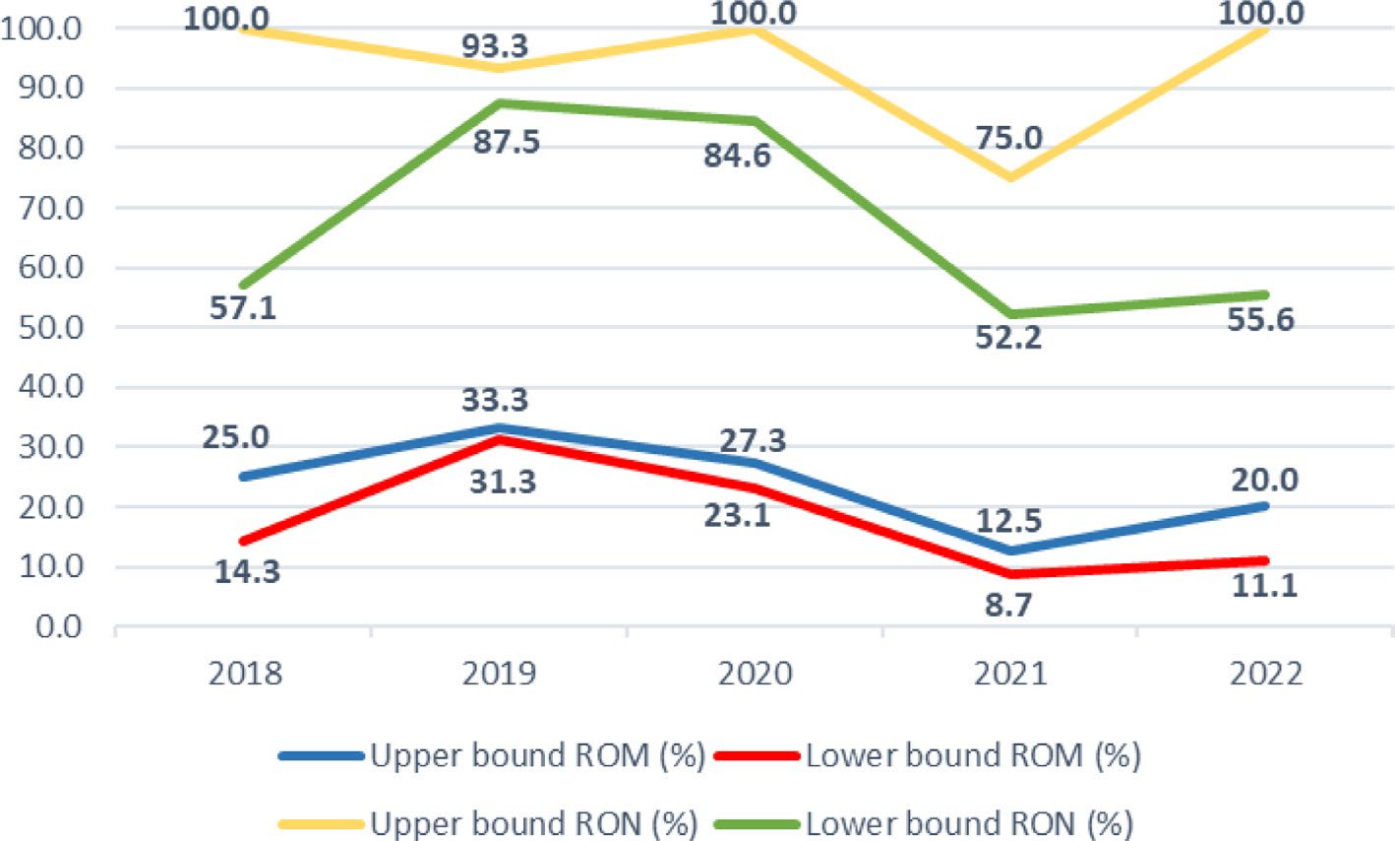
A line chart describes upper‐ and lower‐bound risk of malignancy (ROM) and upper‐ and lower‐bound risk of neoplasm (RON) according to year. [Color figure can be viewed at wileyonlinelibrary.com]

There were three malignant cases in 2018, five in 2019, three in 2020, two in 2021, and three in 2022. There were 12 neoplastic cases in 2018, 14 in 2019, 11 in 2020, 12 in 2021, and 15 in 2022. Figure 2 shows the upper‐ and lower‐bound ROM and RON by year.

### 3.4 Sex Subcategorization

The study cohort consisted of 52 females and 33 males. Histological follow‐ups were available for 37 (71.2%) females and 26 (78.8%) males. The ROM was higher in the male group than in the female group (26.9% and 16.2%, respectively, *p* = 0.353) (Table 3). The highest ROM was in the male group with oncocytic/oncocytoid subtype tumors at 40.0% compared to 14.3% (*p* = 0.338) in female oncocytic/oncocytoid tumors. The basaloid subtype had a higher ROM in the male group (18.8%) than in the female group (16.7%; *p* = 1.000). Pleomorphic adenoma was the most common histological follow‐up in both sex groups. In the male group, the most common malignant neoplasm was carcinoma ex pleomorphic adenoma (*n* = 3), and the most common malignant neoplasms in the female group were adenoid cystic carcinomas (*n* = 2) and carcinomas ex pleomorphic adenoma (*n* = 2) (Table 2).

### 3.5 Age Subcategorization

**FIGURE 3:**
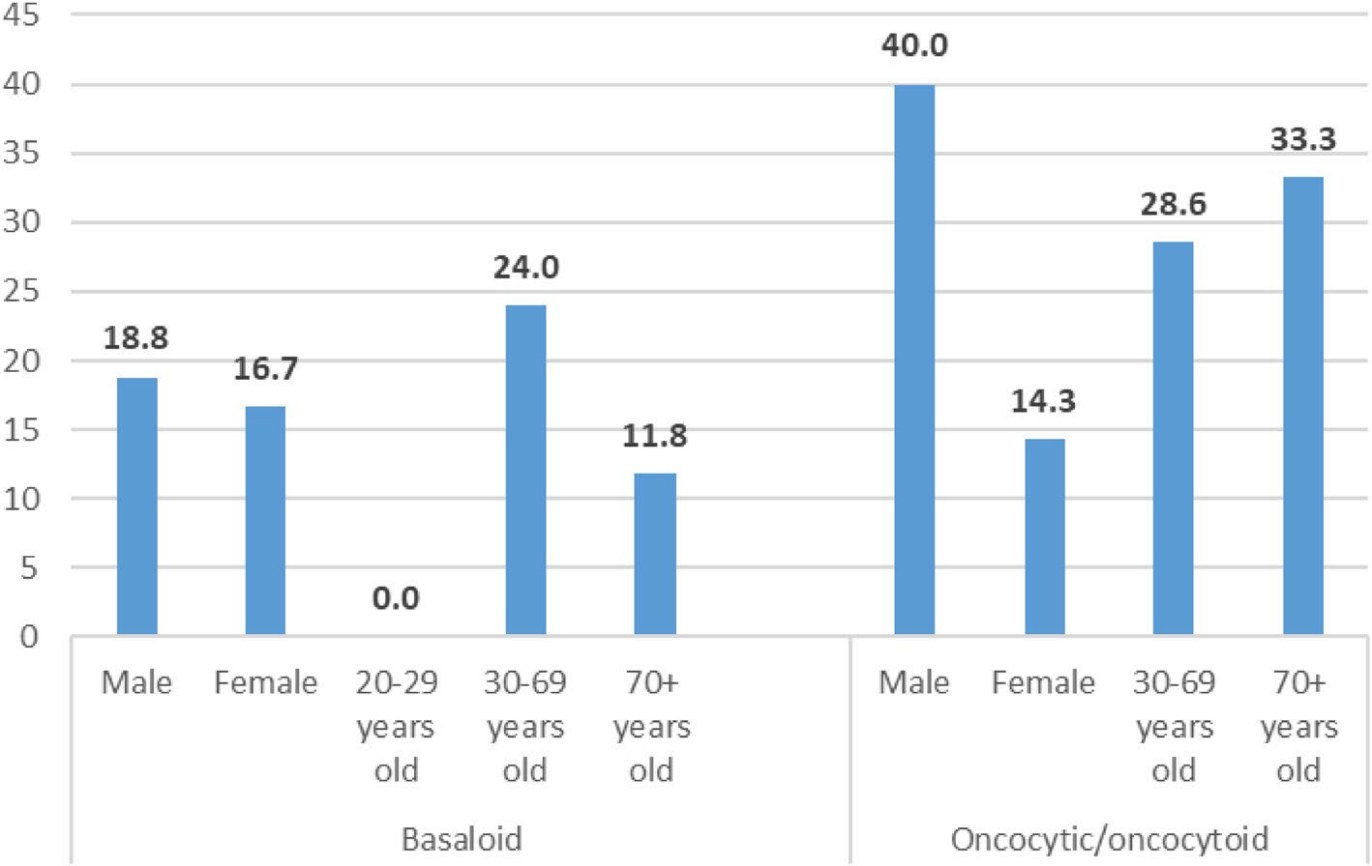
Risk of malignancy based on age, sex, and SUMP subtypes. In the age group 20–29 years old, there were no cases in oncocytic/oncocytoid subtype. [Color figure can be viewed at wileyonlinelibrary.com]

The population was divided into three age groups: 20–29 years old, 30–69 years old, and over 70 years old. In the youngest group, there were five patients, all with surgical follow‐up. In the age group of 30–69 years, there were 36 patients, and 32 (88.9%) cases had surgical follow‐up. In the oldest group, there were 44 patients, but only 26 (59.1%) had surgical follow‐up. In the 20–29 age group, all cases were histologically benign neoplasms, leading to 0% ROM and 100% RON. The age group of 30–69 years had the highest ROM (25.0%). In the oldest age group, there were five malignant cases, and the upper‐bound ROM was 19.2%. In the oncocytic/oncocytoid subtype, the 70+ group had the highest ROM of 33.3%, compared to 28.6% in the 30–69 group (*p* = 1.000). In the basaloid subtype, ROM was 11.8% in the 70+ group and 24.0% in the 30–69 age group (*p* = 0.439). The ROMs and RONs are summarized in Table 3 and in Figure 3. Pleomorphic adenoma was the most common histological diagnosis in all groups. The most common malignant neoplasms were carcinoma ex pleomorphic adenomas (*n* = 4) in the 30–69 age group and mucoepidermoid carcinomas in the oldest group, with three cases (Table 2).

### 3.6 Laboratory Workflow and Patient Management

After the first FNA diagnosed as SUMP, 20 (23.5%) patients had one or more repeat FNAs, based on a decision made by the physician in charge. FNAs were classified according to the MSRSGC into the following categories: 4 (20.0%) FNAs as ND, 1 (5.0%) as AUS, 2 (10.0%) as BN, 15 (75.0%) as SUMP, 1 (5.0%) as SM, and 1 (5.0%) as MN.

Over a 5‐year period, the median time for the pathology laboratory workflow for SUMP cases was 9 (7/13) days from the FNA to the final cytologic diagnosis and 16 (9/22.25) days from the surgical procedure to the final histological diagnosis. The median timeline from the first FNA with SUMP diagnosis to surgical procedure was 77 (46/112.5) days. In the yearly analysis, the laboratory workflow with cytological and histological diagnoses was faster during the years before Covid‐19. With FNAs, the median time for cytological diagnoses was 7 days in 2018 and 8 days in 2019 compared to 8 days in 2020, 10 days in 2021, and 13 days in 2022 (*p* < 0.001). For histological diagnoses, the median time was 15 days in 2018 and 12 days in 2019 versus 12.5 days in 2020, 25.5 days in 2021, and 16.5 days in 2022 (*p* = 0.145) The median timelines over the 5‐year period are shown in Table 4.

**TABLE 4:** Median timelines between the dates of patient managements and pathology laboratory workflows in days and quartiles (min/max).

Grouping	Timeline from FNA to SUMP diagnosis	Timeline from surgical resection to histological diagnosis	Timeline from SUMP FNA to 2. FNA	Timeline from first SUMP FNA to surgical resection	Timeline from last SUMP to surgical resection	Timeline between last FNA to surgical resection
2018	7 (3/11)	15 (11.25/17.5)	85.5 (55.5/118.5)	112 (43.5/180.5)	112 (43/180.5)	112 (43/180.5)
2019	8 (6/9.25)	12 (8.5/18.5)	27 (23.5/33.5)	71 (57.25/88.25)	62 (48/88.25)	62 (48/88.25)
2020	8 (6/10)	12.5 (7.25/23)	60 (44.5/75.5)	73 (66/101)	73 (66/101)	73 (66/101)
2021	10 (7/13)	25.5 (15/37.75)	15 (11/15.5)	65 (38.25/97.75)	65 (37.5/94)	65 (35.75/94)
2022	13 (9/15.5)	16.5 (10/23.25)	88 (47.5/118.75)	84 (41/151)	84 (41/151)	46 (41/84)
2018–2022	9 (7/13)	16 (9/22.5)	44.5 (28.5/97)	77 (46/112.5)	69 (44.5/108)	69 (41.5/101)
Female	9.5 (7/13)	14 (9/24)	44.5 (35/94)	77 (49/98)	69 (46/94)	69 (46/94)
Male	8 (6/13.25)	16 (12/20.75)	29 (21.5/108)	77 (43/138.5)	75 (43/138.5)	65.5 (39.5/112.75)
20–29 years old	10 (7/12)	12 (9/21)	*n* = 0	84 (73/160)	84 (73/160)	84 (73/160)
30–69 years old	8 (6.5/12)	17 (11.5/21.75)	23.5 (18.75/27.5)	82 (53.75/99.5)	75.5 (49/99.5)	75.5 (45.25/99.5)
over 70 years old	10 (7/13)	13.5 (7.25/20.5)	76 (39/112.5)	64 (37.5/131)	54 (37.5/119.5)	49 (37.5/93.5)

Abbreviations: FNA, fine‐needle aspiration; SUMP, salivary gland neoplasm of uncertain malignant potential.

The sex groups had similar access to surgical treatment: the average time from the first FNA with SUMP diagnosis to surgical resection was 77 (43/138.5) days in the male group and 77 (49/98) days in the female group (*p* = 0.911). By age group, patient management was the fastest in the 70+ age group at 64.0 (37.5/131) days, 84 (73/160) days in the 20–29 age group, and 82 (53.75/99.5) days in the 30–69 age group (*p* = 0.544) (Table 4).

## 4 Discussion

Over the 5‐year period, 100 SG‐FNAs from 85 patients were classified as SUMP. Histological verification was available in 69 cases from 63 patients. Five lesions were non‐neoplastic. Pleomorphic adenoma was the most common histological follow‐up, reported in 24 cases. Carcinoma ex pleomorphic adenoma, with five cases, was the most common malignant neoplasm, followed by mucoepidermoid carcinoma in four cases.

In this study, three out of five non‐neoplastic follow‐ups were nodular oncocytic hyperplasia. In another study of 54 cases of SUMP with histological follow‐up, nodular oncocytosis was the most common follow‐up diagnosis after pleomorphic adenoma, with seven cases [14]. The main differential diagnosis of nodular oncocytic hyperplasia is oncocytic neoplasm. Nodular oncocytic hyperplasia is a diagnostic pitfall and is often categorized as SUMP in the MSRSGC.

In this study, Warthin's tumor was a histological diagnosis in six cases. Allison et al.'s study [15] showed that SUMP was the most common MSRSGC category for Warthin's tumor after the BN category: of 324 histologically verified Warthin's tumor cases, 18 were classified as SUMP. One of the diagnostic pitfalls for Warthin's tumor is Warthin‐like mucoepidermoid carcinoma and its differentiation from mucinous metaplastic changes in Warthin's tumor [16]. Another pitfall is that Warthin's tumor with infarction can be diagnostically confused with squamous cell carcinoma, as infarcted Warthin's tumors often contain squamous metaplasia [17]. Warthin's tumor is also a common histopathological diagnosis in the AUS category of the MSRSGC. In our previous study, there were 75 histologically verified AUS cases, and 21 of them were Warthin's tumors [18].

Pleomorphic adenoma is the most common tumor of the salivary glands and is also a typical histological follow‐up in the SUMP category. In the present study and seven other studies that focused on the SUMP category, pleomorphic adenoma was the most common histopathological diagnosis [14, 19, 20, 21, 22, 23, 24]. In a Finnish study of pre‐MSRSGC data focusing on pleomorphic adenoma, 4 of 10 false negative pleomorphic adenoma cases were reclassified as SUMP [25]. Prior to the implementation of the MSRSGC, pleomorphic adenomas were diagnosed as Pap Class 2 and reported as “pleomorphic adenoma” when the cytomorphological features were clear and typical. Cases with some degree of atypia or other abnormalities were classified as Pap Class 3 or 4 and reported as “neoplasm” or “atypia” or “suspicious for malignancy,” depending on the degree of uncertainty [25].

Carcinoma ex pleomorphic adenoma (CXPA) is the malignant counterpart of benign pleomorphic adenoma [26]. It was the most common malignant neoplasm in this study, with five cases. Allison et al.'s study [15] also showed that CXPA was the most common false positive diagnosis for pleomorphic adenoma. The diagnostic accuracy of FNA in identifying elements such as focal metachromatic background and fibrillary matrix in pleomorphic adenoma, including embedded myoepithelial cells, is 70%–90% [26]. Furthermore, Allison et al. study [15] showed that 2.7% of SUMP cases initially diagnosed as pleomorphic adenoma on cytology were histologically confirmed as CXPA after surgical resection. In another study by Klijanienko et al. study [27] on histologically confirmed CXPAs, 50.0% were diagnosed as malignant on cytology, 7.7% as suspicious for malignancy, 38.5% as pleomorphic adenomas, and 3.8% as non‐neoplastic.

Pleomorphic adenoma gene 1 (PLAG1) can be detected in both pleomorphic adenoma and carcinoma ex pleomorphic adenoma. Using PLAG1 immunostaining could potentially reduce the number of pleomorphic adenomas classified as SUMP. Sanchez‐Avila et al.'s study [28] showed that of 45 basaloid SUMP cases, 29 were PLAG1 positive, 6 were equivocal, and 10 were negative. In their study, the use of PLAG1 helped detect pleomorphic adenomas, as PLAG1 was positive in 26 of 30 pleomorphic adenomas. Although PLAG1 expression is not useful for distinguishing between pleomorphic adenoma and carcinoma ex pleomorphic adenoma, it may still aid cytopathologists in differentiating pleomorphic adenoma from other basaloid SUMP tumors, such as basal cell adenoma or adenoid cystic carcinoma [28].

Mucoepidermoid carcinoma was the second most common malignant neoplasm after carcinoma ex pleomorphic adenoma (with four cases) in this study. Miller et al.'s multi‐institutional study [29] showed that of 75 histologically verified mucoepidermoid carcinomas, 26 (34.7%) were cytologically recategorized in the SUMP category. In five other studies that focused on the SUMP category, mucoepidermoid carcinoma was the most common malignant neoplasm [19, 20, 22, 23, 24].

In this study, adenoid cystic carcinoma was the histological follow‐up in three cases. Miller et al.'s multi‐institutional study [29] showed that 11 of the 30 cases of adenoid cystic carcinoma were reclassified as SUMP. Chowsilpa et al.'s study [30] showed that the cytology of adenoid cystic carcinoma may be diagnostically confused with pleomorphic adenoma in the SUMP category: four cases with SUMP basaloid features originally diagnosed as adenoid cystic carcinoma were histologically confirmed to be pleomorphic adenomas. In the present study, basal cell adenoma was the histological diagnosis in six cases. Chowsilpa et al.'s study [30] showed that basal cell adenoma is another common mimic of adenoid cystic carcinoma, and it was misinterpreted in 20% of their cases.

In the present study, acinic cell carcinoma was the histological diagnosis in only two cases. There were 37 cases of histologically verified acinic cell carcinoma in the multi‐institutional study by Miller et al. [29]. Of these, 10 SG‐FNAs were reclassified into the SUMP category. Furthermore, in Wakely et al.'s study [31] with 50 cases of histologically verified acinic cell carcinoma, 10% were classified as SUMP. One of the diagnostic pitfalls for acinic cell carcinomas is oncocytoma, because acinic cell carcinoma's cells can have an oncocytic appearance [32]. In this study, oncocytoma was the histological diagnosis in only three cases. Oncocytomas are classified as SUMP if there is no material for ancillary studies or if adequate clinical and radiological information is not available. Other malignant differential diagnoses of oncocytoma, in addition to acinic cell carcinoma, include oncocytic variants of mucoepidermoid carcinoma, secretory carcinoma, and metastatic carcinoma [5]. In low grade malignancies with low nuclear atypia and bland cytological features, it is not easy to make malignancy diagnosis in certain cases. In addition, entities where capsular invasion is diagnostic, cannot be diagnosed in FNA sample as malignant. In contrast, high grade tumors or tumors with specific immunocytochemistry markers are easy to diagnose as malignant.

The ROM was 23.2% in this study, which is noticeably lower than the MSRSGC [5] reference value (35%). The ROM was probably low due to overdiagnosis in certain benign entities (pleomorphic adenoma, Warthin's tumor, and nodular oncocytic hyperplasia). In the study by Liu et al. [14] focusing on the SUMP category, the ROM was like our results (24.1%). In contrast, six other studies focusing on the SUMP category had a higher ROM, ranging from 31.5% to 40.7% [14, 19, 20, 21, 22, 23, 24]. The reference ROM for the SUMP category did not change from the first MSRSGC edition [6] to the second edition [5]. A meta‐analysis of prospective studies found 36.6% ROM in the SUMP category, which agrees with the MSRSGC reference value [33]. Of note, most of published series originated from university hospital setting [33], but our internal audit (unpublished data) did not show significant differences among cases from university hospital, regional hospitals and community health care centers.

In this study, the number of oncocytic/oncocytoid cases was lower than those with basaloid features, especially where cases of mucoepidermoid carcinoma increased the risk of malignancy in the oncocytic/oncocytoid subtype group. The basaloid subtype group included many cases of benign neoplasms, such as pleomorphic adenoma and basal cell adenoma. The oncocytic/oncocytoid subtype showed 35.0% ROM in this study, while the basaloid subtype showed only 18.4% ROM. In four of six previous studies that subtyped the SUMP category, the oncocytic/oncocytoid subtype showed higher ROM than the basaloid subtype (61.1% and 40.0% [23], 58.8% and 23.0% [21], 52.5% and 36.5% [19], and 41.7% and 30.8% [24], respectively), in agreement with our results. Chowsilpa et al. [22] and Liu et al. [14] showed higher ROM for the basaloid subtype than the oncocytic/oncocytoid subtype (38.5% and 7.7% [22], and 27.6% and 20.0% [14], respectively) (Table 5).

**TABLE 5:** Risk of malignancies of the basaloid and the oncocytic/oncocytoid subtypes in this study and in six previously published studies.

Study	Basaloid subtype		Oncocytic/oncocytoid subtype	
	Cases with surgical follow‐up (*n*)	Risk of malignancy (%)	Cases with surgical follow‐up (*n*)	Risk of malignancy (%)
This study	49	18.4	20	35.0
Arisi et al. [24]	65	30.8	48	41.7
Chowsilpa et al. [22]	13	38.5	13	7.7
Hang et al. [23]	25	40.0	18	61.1
Hang et al. [19]	156	36.5	101	52.5
Liu et al. [14]	29	27.6	25	20.0
Manucha et al. [21]	23	23.0	22	58.8

During the Covid‐19 pandemic, the whole diagnostic process was impaired which also affected salivary gland cytopathology and the SUMP category. The second year of the Covid‐19‐pandemic, 2021, was exceptional in this study; the upper‐bound ROM was only 12.5%, and the upper‐bound RON was the lowest (75.0%). In our previous study focusing on the AUS category, the findings were similar: ROM and RON were the lowest in the Covid‐19 years (2020–2021) [18]. In contrast to our results, Vigliar et al.'s multi‐institutional studies [9, 10] found that the malignancy rate increased from 4.01% to 5.26% during the Covid‐19 pandemic. Furthermore, the studies by Vigliar et al. showed that the number of SG‐FNAs decreased by 59.5% during the lockdown period and by 14.4% during the Covid‐19 post‐lockdown period compared to similar periods in 2019 [9, 10].

The ROM was noticeably higher in the male group than in the female group in this study (26.9% and 16.2%, respectively). Salivary gland tumors are more common in females due to the predominance of benign lesions, while malignant tumors are more frequently diagnosed in males [3]. Worldwide, there were 53,585 new salivary gland cancers in 2020. Of these, 29,694 (55.4%) cases were in males and 23,889 (44.6%) in females [34]. In Finland, during the 10‐year period from 2012 to 2021, there were 384 (52.7%) salivary gland cancers in the male population and 345 (47.3%) in the female population [35]. Lifestyle factors such as smoking, alcohol consumption, and diet could be factors that increase the risk for males. A Canadian case–control study showed that lifestyle factors that are more common in male population such as processed meat, obesity, high alcohol consumption, and exposure to work‐related radiation were associated with an increased risk of salivary gland cancers [36].

In this study, the youngest age group 20–29 did not have any malignant cases; all cases were histologically benign neoplasms. An international multi‐institutional study focusing on pediatric patients (0–21 years) [37] included 22 SUMP cases with histological follow‐up. Of these, 7 were malignant and 15 were benign neoplasms. The upper‐bound ROM was 31.8%, and pleomorphic adenoma was also the most common histological follow‐up in 10 cases [37].

Malignant salivary gland tumors usually appear in the fifth and sixth decades of life [3, 4]. In this study, the 30–69 age group showed the highest ROM of all groups (25.0%). However, all cases of mucoepidermoid carcinoma were in the oldest group, even though a previous study showed that mucoepidermoid carcinoma was more common in patients under 65 years old than in those over 65 [37]. In the age group older than 70 years, only 26 of 44 (59.1%) patients had surgical follow‐up, and the ROM was 19.2%. Underdiagnosis may occur in this age group due to the prioritization of health care [38].

The Covid‐19 pandemic slowed down the whole pathology laboratory workflow, including workflow with FNA, as shown in our previous study of the AUS category [18]. In contrast to our previous study, the laboratory workflow with histological diagnoses was also faster during the years before Covid‐19. However, SUMP diagnoses led to faster surgical resection than AUS diagnoses [18] due to the MSRSGC recommended management [5]. The recommended treatment for the SUMP category is surgery, while for the AUS, it is repeat FNA or surgery. Tenney et al. also found that the average time to surgery was shorter in the SUMP category than in the AUS category. They reported an average of 88.0 days in the SUMP category and 104.8 days in the AUS category [8]. As expected, there were no noticeable differences in timelines between sexes. In some cases, benign salivary gland tumors such as Warthin's tumor can be treated with conservative treatment, especially in the older population [39]. However, surgery is the recommended management for all patients diagnosed with SUMP [5]. In our study, the shortest time for treatment was in the oldest age group, but no statistical significance was found between age groups.

The limitation of this study was its small sample size. The limited number of cases reduces the statistical power of the study, and future research with larger cohorts is needed to confirm these findings.

## 5 Conclusions

This study found a lower ROM than the MSRSGC reference value. Males and those aged 30–69 years had the highest risk of malignancy. The oncocytic/oncocytoid subtype had the highest ROM, mainly due to cases of mucoepidermoid carcinoma. Basaloid benign neoplasms can be overdiagnosed as the basaloid SUMP subtype, leading to a lower ROM. In general, SUMP diagnoses lead to surgical resection relatively quickly. However, the Covid‐19 pandemic slowed down the workflow of the pathology laboratory at our institution. In addition, the second year of the pandemic (2021) showed the lowest ROM of all years studied.

### 5.1 Precis

This study showed 23.2% ROM for the SUMP category, which is lower than the Milan System for Reporting Salivary Gland Cytopathology reference value. The highest ROMs were in the male population and in the 30–69‐year‐old group. The oncocytic/oncocytoid subtype showed a higher ROM than the basaloid subtype.

## Author Contributions

**Henri Lagerstam:** conceptualization, data curation, formal analysis, investigation, methodology, visualization, writing – original draft, and writing – editing. **Erkka Tommola:** data curation, formal analysis, and writing – editing. **David Kalfert:** formal analysis, methodology, and writing – editing. **Saara Kares:** data curation and writing – editing. **Heini Huhtala:** formal analysis, methodology, writing‐editing. **Ivana Kholová:** conceptualization, data curation, formal analysis, investigation, methodology, project administration, resources, visualization, supervision, validation, writing – original draft, and writing – editing.

## Conflicts of Interest

The authors declare no conflicts of interest.

## Data Availability

The data that support the findings of this study are available from the corresponding author upon reasonable request.
